# Diagnostic and Prognostic Performance of Neurofilaments in ALS

**DOI:** 10.3389/fneur.2018.01167

**Published:** 2019-01-18

**Authors:** Koen Poesen, Philip Van Damme

**Affiliations:** ^1^Department of Neurosciences, Laboratory for Molecular Neurobiomarker Research, KU Leuven, Leuven, Belgium; ^2^Laboratory Medicine, University Hospitals Leuven, Leuven, Belgium; ^3^Laboratory of Neurobiology, Department of Neurosciences, KU Leuven and Center for Brain & Disease Research VIB Leuven, Leuven, Belgium; ^4^Department of Neurology, Neuromuscular Reference Centre, University Hospitals Leuven, Leuven, Belgium

**Keywords:** amyotrophic lateral sclerosis, frontotemporal dementia, neurofilament, cerebrospinal fluid, serum, plasma, NfL, pNfH

## Abstract

There is a need for biomarkers for amyotrophic lateral sclerosis (ALS), to support the diagnosis of the disease, to predict disease progression and to track disease activity and treatment responses. Over the last decade multiple studies have investigated the potential of neurofilament levels, both in cerebrospinal fluid and blood, as biomarker for ALS. The most widely studied neurofilament subunits are neurofilament light chain (NfL) and phosphorylated neurofilament heavy chain (pNfH). Neurofilament levels are reflecting neuronal injury and therefore potentially of value in ALS and other neurological disorders. In this mini-review, we summarize and discuss the available evidence about neurofilaments as diagnostic and prognostic biomarker for human ALS.

## Introduction

Amyotrophic lateral sclerosis (ALS) is a neurodegenerative disorder primarily affecting the motor system network, giving rise to progressive muscle weakness in the limbs, the bulbar region, but also of the respiratory muscles. Survival is typically between 2 and 5 years after disease onset, but in about 15% of patients a slower disease progression is present (1). The most important extramotor manifestations of the disease include behavioral changes, executive dysfunction and language problems, reminiscent of frontotemporal dementia.

As of today, the diagnosis of ALS remains based on clinical judgement and requires a combination of signs of upper and lower motor neuron involvement in a patients with progressive muscle weakness, without alternative explanation for the presenting symptoms and signs (2). Despite efforts to make the diagnostic criteria more sensitive (3, 4), the diagnostic delay remains about 10–12 months after symptom onset (5). The current clinical criteria also do not discriminate between different subtypes of ALS, although they may have very different disease trajectories. Combinations of clinical parameters allow to predict disease progression and survival in ALS patients, but they do not reflect the underlying biological processes (6).

Biomarkers, which reflect hallmarks of the disease, may not only aid in the diagnostic algorithm of ALS, but could also be of value in defining homogeneous subgroups of patients. Potentially, they could also be helpful to track disease progression and treatment responses (7). Neurofilaments (NF) have been studied extensively in different neurological conditions, and are considered to be useful as marker of acute and chronic neuronal injury (8). Neurofilaments are intermediate filaments of 10 nm in neurons, composed of heteropolymers of different subunits, neurofilament light chain (NfL), neurofilament medium chain (NfM), and neurofilament heavy chain (NfH) (9). Phosphorylation and O-glycosylation are believed to be important for NF assembly (9) and especially NfM and NfH undergo these posttranslational modifications. NF are highly expressed in neurons, provide structural support for neurons and determine axon caliber and conduction velocity (10). Mutations in the genes encoding NfH and NfL can cause the inherited neuropathy Charcot-Marie-Tooth disease (11), inframe deletions or insertions in the side arm domain or C-terminal tail domain of NfH have also been linked to ALS (12). Neurofilamentous abnormalities and elevated NF levels are not restricted to ALS. However, NF have been implicated in the pathogenesis of ALS for more than 2 decades (13). In post mortem spinal cord of ALS patients, accumulations of NF are seen in the perikaryon and axons of motor neurons (14) and motor neurons display reduced NfL mRNA levels (15). Overexpression of NfH causes a motor axonopathy with NF inclusions in mice, which can be rescued by NfL overexpression (16), suggesting that an imbalance between the relative expression levels of the different NF subunits may be important. In line with this hypothesis, reducing the NfL levels and overexpression of NfH levels in the SOD1 mouse model of ALS, increased the lifespan of these animals (17, 18). In this model of ALS, the degeneration of motor neurons is accompanied by a progressive rise in blood NF levels, and these levels have been shown to be able to capture treatment responses (19, 20).

In this review, we will give an overview of the current knowledge about the diagnostic and prognostic value of NF levels in cerebrospinal fluid and blood for human ALS.

## Available Methods to Measure Neurofilaments Levels

Numerous studies employed in house developed assays or commercial “for research use only” ELISAs for NF measurements (20–27). Although the precision and recovery profile of such kits was acceptable (Table 1), the analytical sensitivity in terms of limit of detection and limit of quantification was insufficient to precisely detect NF levels in CSF of controls or in blood of most patients with ALS (30). Using the same antibodies against NfL, novel technologies including electrochemiluminescence (ECL) and Single Molecule Array (SIMOA) enabled to precisely and sensitively quantify NfL in CSF and blood (22, 29, 40). Furthermore, an improved ELISA assay allowed to accurately quantify pNfH in blood and CSF of patients with ALS (39).

**Table 1:** Analytical and diagnostic performance of NfL and pNfH assays.

	**Capture antibody epitope**	**Calibrator**	**Analytical performance**					**Diagnostic performance (ALS vs. mimics)**					
			**LOD**	**LOQ**	**CV_**wr**_**	**CV_**rr**_**	**Recovery**	**Sens**	**Spec**	**PPV**	**NPV**	**LR+**	**LR-**
**NfL**			**pg/mL**		**%**	**%**	**%**	**%**	**%**	**%**	**%**		
Gothenburg (Blennow lab) ELISA (21)	Core domain (aa 93-396) of full length recombinant NfL (Origene)	Not disclosed	-	78^a,b^	8^a,b^	13^a,b^	80 - 109^a,c^	Not addressed					
NF-light ELISA (22) (Uman Diagnostics)	Core domain (aa 92–396) (NF-L mAb 47:3 and NF-L mAb 2:1 having nonsterical overlapping epitopes) (28)	Purified bovine spinal cord NfL (Progen Biotechnik GmbH)	^−^	78^(29),*c,e*^	1.5 ^a,c^	17.4^a,c^	-	78^(23),*c*^ 77^(30),*c*^ 89^(31), *c*^ 96.2^c,(32)^	85 88 89 56.3	92 95 - -	64 56 - -	3.9 - - -	- - - -
Simoa NF-light digital immunoassay (29) (Quanterix)			0.038^a,o^	0.62^(29),*c,e*^	6.6	17.0	118.5^c,o^ 90.7^d,o^	100^d,(31)^ 85.5^d,(33)^	84 77.3	- 91.4	- 65.4	- -	- -
NFL ECL assay (29)			-	15.6^c,e^	9.2 3.6^p^	14.8 6.6 ^p^	-	79^(34),*d*^	84-90^f^	98	34	-	-
**pNfH**													
Nijmegen pNfH ELISA (35)	Low to highly phosphorylated NFH (Anti-SMI35 (or 03-44) Ab via Sternberger Monoclonals Incorporatedand later on via Covance Research Products) (36) Cross-reacting for 7.8% with NFM (25)	Bovine NFHp35 standard (ICN, Burlingame, CA)						72^c^	80 ^c^	-	-	-	-
London pNfH ELISA (25)		HPLC-purified bovine NfH (Affiniti Research	200^c,g^		10.6 ^c^ 7.9^c,(26)^	23^c^ 9.76^h^-14.08^I,c,(26)^	119^(26)^		CSF levels of NfHSMI35 were five times higher in patients with ALS (1.7 ng/mL) than in controls (37)				
pNfH ECL assay (38)		Bovine NfH (USBiological)	(0.6) 24 ^g^		4.8^c^	8.4^c^	84.5-93.2^c^	-	-	-	-	-	-
Gainesville pNfH ELISA (26, 27)	220 kDa Form of NFH isolated from bovine Spinal cord [affinity purified chicken anti pNfH via EnCor Biotechnology (Alachua, Florida)], no obvious Reacting with of NFM or other lower molecular weight material (27)	In house purified bovine NfH	400^g^		2.96 ^c,h^,	6.67^c,h,j^	-	-	-	-	-	-	-
Euroimmun pNfH ELISA		Purified bovine pNfH (EnCor Biotechnology Inc., Gainesville, Florida, USA)	69^c,n^	150^c,n^	5.50^a,o^ 6.90 ^c,n^	5.95^a,o^ 9.17 ^c,n^	-	92.9^c,(39)^ 100^c,m, (32)^ 71.8^d,(39)^	96.0 68.8 85.2	98.7 - 93.8	80.0 - 48.9	23.3 - 4.8	- - -
Jacksonville pNfH ELISA (20)	Bovine pNfH purified from spinal cord (purified AH1 monoclonal antibody)	Bovine pNhH purified from spinal cord	31.25^g,k^			8.5-12.5^l^	-	First paper to show higher levels of plasma pNfH in ALS than in healthy controls					
Biovendor pNfH ELISA (24)	Not publically available		23.5^g,o^	-	4.5^a,b,o^	-	98.8^c,o^ 95.8^d,o^	84.4^c,m^ 83^c,(30)^ 90.7 ^c,(23)^	93.5 ^c,m^ 80 88.0	- 93 95.8	- 62 75.9	- - 7.6	- - -

*a, protocol not (fully) disclosed/available; b, matrix not disclosed; c, in CSF; d, in serum; e, value below lowest calibrator reaching accuracy of 80-120% and CV% ≤ 20% (which is most closely related to the definition of LOQ); f, <6 m and >6 m disease duration, respectively; g, signal of blank + 3 times the standard deviation, considered as limit of blank within current review; h, high pNfH sample; i, low pNfH sample; j, 9 consecutive days for 12 samples; k, serially diluted bovine pNfH; l, standards run 16 times on each of 7 days; m, in disease controls also including ALS mimics; n, in house data (not published) via the “Verification of Performance for Precision and Trueness; Approved Guideline-Second Edition”. CLSI document EP15-A2.; o, manufacturer's data - p, low NfL levels ranging from 9.91 pg/mL to 72.8 pg/mL. aa, amino acids; CVwr, within-run or intra assay coefficient of variance; CVrr, between-run or inter assay coefficient of variance; ECL, Electrochemiluminescence; ELISA, Enzyme-Linked Immunosorbent Assay; LOD, limit of detection; LOQ, limit of quantification; LR, likelihood ratio for abnormal (+) or normal (-) result; NFM, neurofilament medium chain; NPV, negative predictive value; PPV, positive predictive value; sens, sensitivity; spec, specificity*.

## Diagnostic Value of Neurofilaments

It is already known for more than 2 decades that NF levels are roughly 5–10 times higher in ALS patients compared to healthy controls (41). Numerous studies since then, have shown that NF levels are increased in patients with ALS, not only in CSF, but also in serum or plasma (42). As NFs are produced by neurons, the serum/plasma levels are 10 fold lower compared to CSF levels.

Several studies showed that NfL and pNfH are elevated in CSF and serum/plasma in patients with ALS (20, 23, 30–32, 35, 37–39, 43–55). There is a good correlation between NF levels in CSF and in blood, and this is the case for NfL and pNfH (34, 39, 40). Nevertheless, the diagnostic performance was found to be better in CSF compared to blood (39, 54). Most studies compared ALS patients to healthy controls, only few studies tested the diagnostic performance in comparison to ALS mimicking disorders (23, 30, 31, 39). The sensitivity and specificity for ALS was better for pNfH than for NfL in studies comparing both neurofilament subunits (23, 32, 39). Even though there is considerable elevation in NF in some of the ALS mimicking disorders, the diagnostic accuracy to detect ALS is still good. The diagnostic performance of NfL and pNfH assays is shown in Table 1. One study suggested that the discrimination from disease controls improved by using the CSF pNfH/complement C3 ratio (24). For implementation in the routine clinical practice, assay standardization, and characterization, and independent validation of the cut-offs are required. Indeed, the development of reference methods for NF measurements, e.g., by means of mass spectrometry (56, 57), and of certified reference materials for traceability of the calibrators and to demonstrate commutability among the different assays should be encouraged (58). Independent evaluation of the performance characteristics of the NF assays enables the public availability of data on the analytical quality of the different commercially available assays. Furthermore, automation of immunoassay facilitates single measurements with similar precision profiles as duplicate measurements in manually performed ELISAs, the former significantly reducing the implementation costs for patients (59). As the range of NF levels in ALS mimicking disorders is rather wide, the robustness of reported cut-offs might be challenged by the rather low number of ALS mimicking disorders included in most studies (23, 39). Multicenter studies are warranted to establish universally applicable cut-offs for NF.

Importantly, the increase in NF is already measurable early in the disease course (23, 31, 40). A recent study showed that NfL levels increase already several months prior to symptom onset in *SOD1* mutation carriers (60). NFs are elevated in sporadic and familial ALS patients, although slightly lower in confirmed *SOD1* cases (43) and higher in *C9orf72* positive patients (51).

The neuroanatomical correlate of elevated NF levels in ALS is not entirely clear. Both NfL and pNFH correlate with the extent of clinical upper and lower motor neuron involvement (23), although pNfH levels correlate better with lower motor neuron involvement and NfL levels better with upper motor neuron involvement (23, 34). An imaging study revealed that NfL levels in CSF correlate with the extent of corticospinal tract involvement on DTI (48).

## Prognostic Value of Neurofilaments

The levels of both NfL and pNfH have been shown to correlate with parameters of disease severity, such as the decline on the ALS functional rating scale-revised or ALSFRS-R (23, 30, 34). They also predict survival of ALS patients, with higher NF levels being unfavorable. In Cox regression analyses both NfL and pNfH have been shown to be independent predictors of survival, when taking other prognostic factors into account (30, 34, 45, 61). Patients with very long survival typically have low levels of NFs (23, 53). The predictive value of NFs is present when using both CSF and blood samples. As higher NF levels are associated with a faster disease progression in typical ALS patients, NF levels could theoretically be used to stratify patients in clinical trials. However, data on this topic are currently lacking.

The difference in disease progression between different clinical subtypes of ALS is not always reflected in NF levels. Patients with *C9orf72* ALS have been reported to have higher pNfH CSF levels (51), but further studies on NF levels are needed in different motor neuron disease subtypes. In patients with primary lateral sclerosis (PLS), the levels can also be increased, but mostly to a lesser extent (30, 31, 34). ALS patients with cognitive/behavioral impairment or comorbid FTD have a worse outcome (62, 63), but if this is reflected in NF levels requires further study (64). The unfavorable outcome of patients with bulbar onset or respiratory onset ALS may not be reflected in NF levels.

## Value of Neurofilaments to Track Treatment Response?

NFs may not only have value to help with the diagnosis and prediction of disease severity in ALS, they may also become of value to track the response to treatments. As marker of neuronal injury it is anticipated that neuroprotective treatments would result in lower NF levels. For ALS, there are no studies in patients that report a treatment response on NF levels at present. Whether the effect of riluzole on survival can be captured by measuring NF levels remains unknown. On the other hand, a recent study using rodent mutant SOD1 models, showed a clear survival benefit of treatment with antisense oligonucleotides, which was accompanied by a reduction in serum pNfH levels (65). In addition, in other neurological disorders, such as multiple sclerosis, NFs levels reflect the effect of disease-modifying therapies (66).

In patients with ALS, it is know that NFs levels are relatively stable during the course of the disease in many patients (51, 67). However, there is some evidence that the levels may increase during the first phase of the disease (53). This is backed up by data from a recent study in *SOD1* mutation carriers, which showed that the levels slowly increase up to 12 months prior to symptom onset and can continue to rise the months following symptom onset (60). The NF levels also correlate with the number of body regions affected by ALS and the ALS progression rate (23, 34), suggesting that they reflect the extent and rate of motor neuron degeneration. Several cross-sectional studies have reported a negative correlation of NF levels with survival (30, 34, 53). This may suggest that the levels drop slightly in later disease stages, although there certainly is a bias introduced by the enrichment for patients with a longer survival at later time points. Longitudinal sampling shows a tendency to lower levels upon follow up, especially in fast-progressing patients (67).

## Conclusion

Evidence is emerging that NF levels can become valuable biomarker for ALS, both for diagnosing ALS, for predicting outcome, and potentially for the monitoring of treatment effects. The CSF pNfH level seems to be the most accurate diagnostic marker, but both pNfH and NfL serum or plasma measurements perform good to predict survival and disease progression. Further research is needed to establish the value of NF levels for stratification and for disease monitoring in clinical trials.

## Author Contributions

All authors listed have made a substantial, direct and intellectual contribution to the work, and approved it for publication.

### Conflict of Interest Statement

The authors declare that the research was conducted in the absence of any commercial or financial relationships that could be construed as a potential conflict of interest.
